# Degalactosylated Whey Protein Suppresses Inflammatory Responses Induced by Lipopolysaccharide in Mice

**DOI:** 10.3389/fnut.2022.852355

**Published:** 2022-04-29

**Authors:** Toshio Inui, Namiko Kawamura, Riho Nakama, Akio Inui, Goro Katsuura

**Affiliations:** ^1^Saisei Mirai Cell Processing Center, Osaka, Japan; ^2^Kobe Saisei Mirai Clinic, Kobe, Japan; ^3^Inui Immunotherapy Clinic, Osaka, Japan; ^4^Drug Discovery of Next-Generation GcMAF, Kagoshima University Graduate School of Medical and Dental Sciences, Kagoshima, Japan; ^5^Pharmacological Department of Herbal Medicine, Kagoshima University Graduate School of Medical and Dental Sciences, Kagoshima, Japan

**Keywords:** whey protein, degalactosylation, lipopolysaccharide, cytokines, mice, inflammation, macrophage

## Abstract

The effects of degalactosylated whey protein on lipopolysaccharide (LPS)-induced inflammatory responses in mice were observed in comparison with intact whey protein. Intraperitoneal administration of both intact and degalactosylated whey proteins for 5 days did not affect body weight and food intake in mice. On day 6, intraperitoneal administration of LPS induced a marked decrease in body weight 4 h later. The LPS-induced decrease in body weight was significantly suppressed by the administration of degalactosylated whey protein, but not intact whey protein. Administration of LPS also significantly increase plasma tumor necrosis factor-α (TNF-α) and interleukin-1β (IL-1β) levels, which were significantly suppressed by the administration of degalactosylated whey protein, but not intact whey protein. Moreover, the application of degalactosylated whey protein to RAW264.7 cells significantly reduced mRNA expression of toll-like receptor 4 (TLR4) and significantly increased mRNA expression of mitogen-activated protein kinase phosphatase-1 (MKP-1). The marked increased expression of TNF-α and IL-1β in response to LPS in RAW264.7 cells was significantly suppressed by the application of degalactosylated whey protein. These results suggest that degalactosylated whey protein suppresses the effects of LPS in part by decreasing in TLR4 and increasing in MKP-1.

## Introduction

The protein fraction of milk contains many valuable nutritional components and biologically active substances (1). Milk contains two primary sources of protein, casein and whey (2). Whey is the liquid remaining after precipitation and removal of milk casein curd during the manufacture of cheese, and recognized as a functional food with nutritional applications and health benefits, including disease prevention and treatment (2, 3). Whey comprises beta-lactoglobulin, alpha-lactalbumin, bovine serum albumin, lactoferrin, immunoglobulins, lactoperoxidase enzymes, glycomacropeptides, lactose, and minerals (3), and has antioxidant, antihypertensive, antitumor, antiviral, hypolipidemic, and anti-bacterial effects *in vitro* and *in vivo* (2). Several lines of evidence suggest that whey protein has immunoregulatory effects (4–7). For example, animal studies revealed that whey protein reduces the levels of proinflammatory cytokines, such as tumor necrosis factor-α (TNF-α) and interleukin-1β (IL-1β), and increases the levels of anti-inflammatory cytokines, such as interleukin-4 and interleukin-6 (8–11), making whey protein a promising therapeutic candidate for immunomodulation in different diseases (11). The precise mechanisms underlying the immunomodulatory effects of whey protein, however, are not clear. Moreover, the effects of whey protein on the actions of lipopolysaccharide (LPS) are not examined.

Protein glycosylation is involved in multiple biological processes, such as cell recognition, differentiation, development, signal transduction, and immune responses (12). Glycoproteins are present in various forms, e.g., enzymes (13, 14), peptide hormones, antibodies, lectins, membrane bound proteins, collagen, and fibronectin, to induce biological activities (15–20). Deglycosylation converts biologically inactive proteins to biologically active proteins. For example, deglycosylated melanin-concentrating hormone receptor is fully active for ligand binding and signal transduction (21).

In the present study, we examined the immunomodulatory effects of degalactosylated whey protein on the inflammatory responses to LPS in mice. Our findings revealed that the functional benefits of the degalactosylation of whey protein in converting inactive protein to active protein in suppressing the inflammatory responses to LPS, of which possible mechanisms are partially attributed to decrease in toll-like receptor 4 (TLR4) and increase in mitogen-activated protein kinase phosphatase-1 (MKP-1).

## Materials and Methods

### Experimental Animals

Male C57BL/6J mice (6 weeks old) were obtained from CLEA Japan, Inc. (Tokyo, Japan) and individually housed in plastic cages under a 12:12 h light/dark cycle (lights turned on at 7 am) at room temperature (23 ± 1°C). The animals were given *ad libitum* access to water and food (CE-2; CLEA Japan, Inc.). Animals were used in the experiments at 10 weeks of age. Eight to twelve mice were used in each group. All experiments were performed in accordance with the guidelines established by the Institute of Laboratory Animal Science Research Support Center at Kagoshima University and the United States National Institutes of Health Guide, and approved by the Kagoshima University Institutional Animal Care and Use Committee for the care and use of laboratory animals. Every effort was made to optimize the comfort of the animals and to minimize their use.

### Preparation of Degalactosylated Whey Protein

Whey protein was obtained from Yotsuba Milk Products Co., Ltd. (Sapporo, Japan). The whey protein was first treated with enzyme as described previously (22). Briefly, 1 mg of whey protein was dissolved in 1 ml of 50 mM sodium phosphate buffer (pH 7.0) and incubated with 65 mU of β-D-galactosidase (from Escherichia coli; WAKO Pure Chemical Industries, Ltd., Osaka, Japan) at 37^°^C for 1 h. The reaction mixture was heated at 60^°^C for 10 min to inactivate the enzyme. The protein concentrations were determined using a Pierce^®^ BCA protein assay kit (Thermo Fisher Scientific Inc., Waltham, MA, United States).

### *In vivo* Experimental Schedule in Mice

**FIGURE 1:**
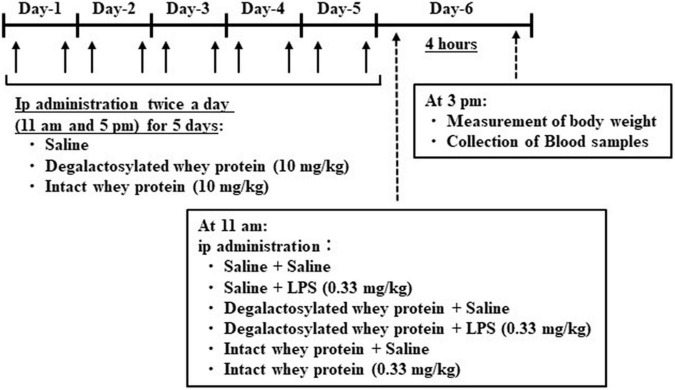
Schematic representation of *in vivo* experimental schedule in mice.

Intact and degalactosylated whey proteins (10 mg/kg) were intraperitoneally administered twice a day at 11 a.m. and 5 p.m. for 5 days in mice (Figure 1), according to a previous report (9, 23). Body weight and food intake were measured every day for 5 days as same as our previous reports (24, 25). On day 6, the intact and degalactosylated whey proteins were intraperitoneally administered with LPS (0.33 mg/kg). According to the previous report showing that LPS-induced sickness behavior and elevation of plasma levels of pro-inflammatory cytokines, IL-1β and TNF-α, in mice appears at 2 h after LPS injection and persists for 7 h (26–28), blood samples were collected from the retroorbital vein 4 h later under isoflurane anesthesia and immediately transferred to tubes containing EDTA (10 μl of 0.2 M EDTA/tube) and aprotinin (0.1 mg/tube, Merck KGaA, Darmstadt, Germany). The blood samples were centrifuged at 3,000 × g for 5 min at 4°C, and the plasma was separated and stored at –80°C until assayed. Body weight changes were also measured at 4 h after LPS administration.

### Enzyme-Linked Immunosorbent Assay

Using 100 μl of plasma, plasma levels of tumor necrosis factor-α (TNF-α) and interleukin-1β (IL-1β) were measured using commercially available kits, TNF-α kit (Mouse TNF-alpha Quantikine ELISA Kit, R&D Systems, MN, United States) and IL-1β kit (Mouse IL-1 beta/IL-1F2 Quantikine ELISA Kit, R&D Systems), respectively.

### Cell Cultures

The mouse macrophage cell line RAW264.7 (ECACC 91062702, European Tissue Culture Collection, London, United Kingdom) was cultured in Dulbecco’s Modified Eagle Medium (DMEM; Thermo Fisher Scientific Inc.) containing 10% fetal bovine serum (Thermo Fisher Scientific Inc.) and 1% antibiotic-antimycotic solution (Thermo Fisher Scientific Inc.), seeded in 6-well plates (5 × 10^5^ cells/ml/well), and maintained in 95% humidified air and 5% CO2 at 37°C for 3–4 days, as described previously (29). Cells were incubated with degalactosylated whey protein (100 μg mg/ml) for 3 h to evaluate the effects of degalactosylated whey protein on the mRNA expression of TLR4 and MKP-1. Moreover, to examine the effects of degalactosylated whey protein on the LPS-induced increase in the mRNA expression of TNF-α and IL-1β, cells were first incubated with degalactosylated whey protein (100 μg mg/ml) for 3 h, and then with LPS (5 ng/ml) and degalactosylated whey protein (100 μg mg/ml) for another 3 h.

### Reverse Transcription-Polymerase Chain Reaction

The mRNA levels were measured by quantitative real-time reverse transcription-polymerase chain reaction (RT-PCR) (29, 30). Following the treatment, the cells were lysed and total RNA was isolated using RNeasy Mini Kit (QIAGEN, Hilden, Germany), and cDNA was synthesized using Verso cDNA Synthesis Kit (Thermo Fisher Scientific Inc.). Aliquots of diluted cDNA were amplified with FastStart SYBR Green Master (Roche Applied Science, Mannheim, Germany) in a final volume of 25 μl on Thermal Cycler Dice Real Time System (TAKARA BIO INC., Shiga, Japan). The RT-PCR schedule was 95°C for 10 min followed by 40 cycles of 5 s at 95°C, 30 s at 60°C and 1 cycle of 15 s at 95°C, 30 s at 60°C and 15 s at 95°C. The samples were run in duplicate. All gene-specific mRNA expression values were normalized against the internal housekeeping gene, glyceraldehyde-3-phosphate dehydrogenase (GAPDH). Primers were as follows: GAPDH (sense TGCACCACCAACTGCTTAGC, antisense GGATGCAGGGATGATGTTCTG); TLR4 (sense GCTTTCA CCTCTGCCTTCAC, antisense AGGCGATACAATTCCACC TG); MKP-1 (sense GGCCAGCTGCTGCAGTTTGAGT, antisense AGGTGCCCCGGTCAAGGACA); TNF-α (sense GTG GAACTGGCAGAAGAG, antisense CCATAGAACTGATG AGAGG); IL-1β (sense CTGTGTCTTTCCCGTGGACC, antisense CAGCTCATATGGGTCCGACA).

### Statistical Analysis

Data are expressed as mean ± SEM. Statistical analysis of the data was performed by ANOVA followed by the Tukey-Kramer test. Statistical significance was defined as *P* < 0.05.

## Results

### Effects of Intraperitoneal Administration of Degalactosylated Whey Protein for 5 Days on Body Weight and Food Intake in Mice

**FIGURE 2:**
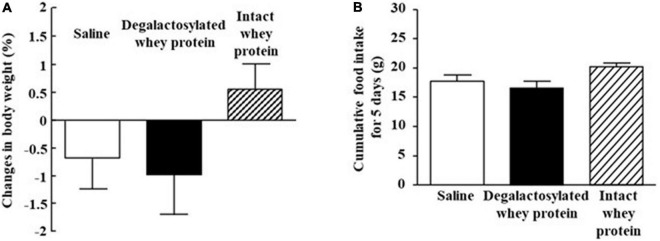
Effects of intraperitoneal administration of degalactosylated whey protein for 5 days on body weight and food intake in mice. **(A)** Body weight changes (%) in mice after intraperitoneal treatment of intact and degalactosylated whey proteins (10 mg/kg) for 5 days. **(B)** Cumulative food intake in mice intraperitoneally treated with intact and degalactosylated whey proteins (10 mg/kg) for 5 days. Results are expressed as mean ± SE for 8–12 mice.

Intraperitoneal administration of both intact and degalactosylated whey proteins (10 mg/kg) for 5 days did not affect body weight in mice (Figure 2A). In addition, intraperitoneal administration of both intact and degalactosylated whey proteins for 5 days did not influence cumulative food intake for 5 days in mice (Figure 2B).

### Effects of Intraperitoneal Administration of Degalactosylated Whey Protein for 5 Days on Body Weight Loss Induced by Intraperitoneal Administration of Lipopolysaccharide in Mice

**FIGURE 3:**
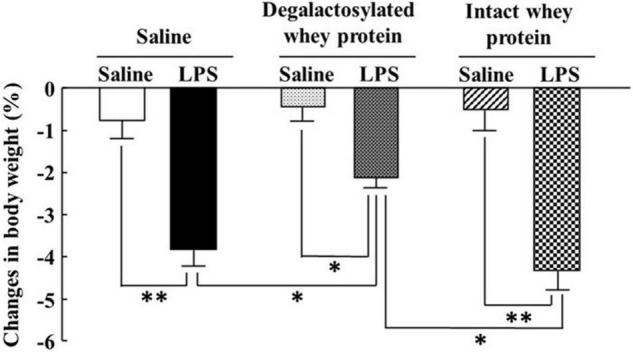
Effects of intraperitoneal administration of degalactosylated whey protein for 5 days on body weight loss induced by intraperitoneal administration of LPS in mice. Body weight changes (%) in mice at 4 h after intraperitoneal administration of LPS (0.33 mg/kg) following intraperitoneal administration of intact and degalactosylated whey proteins (10 mg/kg) for 5 days. Results are expressed as mean ± SE for 8–12 mice. *p < 0.05, ^**^p < 0.01.

Intraperitoneal administration of LPS (0.33 mg/kg) induced a marked decrease in body weight 4 h later, which was significantly suppressed by intraperitoneal administration of degalactosylated whey protein (10 mg/kg) in mice pretreated with degalactosylated whey protein to 62% of the body weight loss induced by LPS treatment in saline-pretreated mice (Figure 3). Intact whey protein did not influence LPS-induced body weight loss in mice (Figure 3).

### Effects of Intraperitoneal Administration of Degalactosylated Whey Protein for 5 Days on the Increase in Plasma Tumor Necrosis Factor-α and Interleukin-1β Levels Induced by Intraperitoneal Administration of Lipopolysaccharide in Mice

**FIGURE 4:**
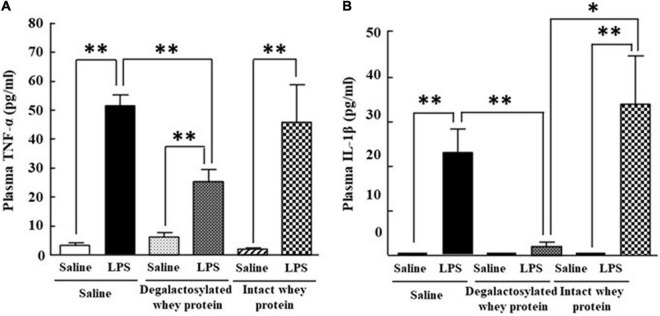
Effects of intraperitoneal administration of degalactosylated whey protein for 5 days on increases in plasma TNF-α and IL-1β levels induced by intraperitoneal administration of LPS in mice. **(A)** Changes in plasma TNF-α levels at 4 h after intraperitoneal administration of LPS (0.33 mg/kg) following intraperitoneal administration of intact and degalactosylated whey proteins (10 mg/kg) for 5 days in mice. **(B)** Changes in plasma IL-1β levels at 4 h after intraperitoneal administration of LPS (0.33 mg/kg) following intraperitoneal administration of intact and degalactosylated whey proteins (10 mg/kg) for 5 days. Results are expressed as mean ± SE for 8–12 mice. *p < 0.05, ^**^p < 0.01.

Intraperitoneal administration of LPS (0.33 mg/kg) in mice induced a marked increase in plasma TNF-α and IL-1β levels 4 h later (Figures 4A,B). The increase in the plasma TNF-α and IL-1β levels induced by LPS was significantly suppressed by intraperitoneal administration of degalactosylated whey protein (10 mg/kg) in mice pretreated with degalactosylated whey protein (10 mg/kg) to 54 and 10%, respectively, of the levels induced by LPS treatment in saline-pretreated mice, respectively (Figures 4A,B). Intact whey protein (10 mg/kg) did not suppress the increase in these plasma cytokine levels induced by intraperitoneal administration of LPS (Figures 4A,B).

### Effects of Degalactosylated Whey Protein on the mRNA Expression of Toll-Like Receptor 4 and Mitogen-Activated Protein Kinase Phosphatase-1, and Lipopolysaccharide-Induced mRNA Expression of Tumor Necrosis Factor-α and Interleukin-1β in RAW264.7 Cells

**FIGURE 5:**
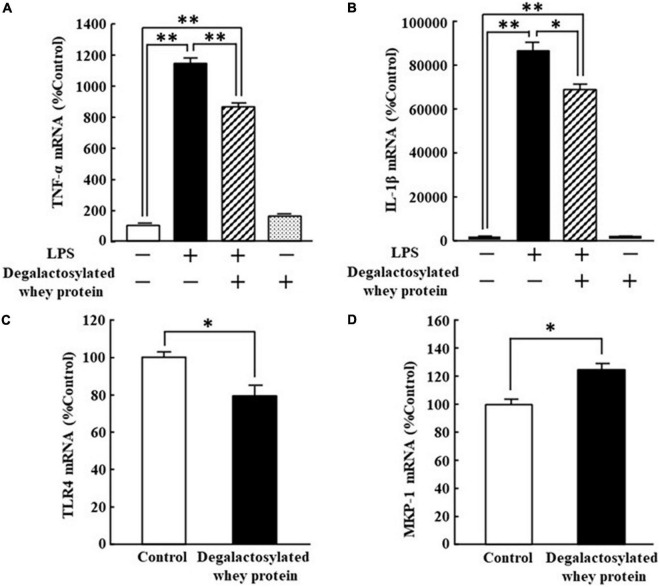
Effects of degalactosylated whey protein on mRNA expression of TLR4 and MKP-1, and LPS-induced mRNA expression of TNF-α and IL-1β in RAW264.7 cells. **(A)** Effects of 3-h preincubation with degalactosylated whey protein (100 μg mg/ml) on mRNA expression of TNF-α induced by LPS (5 ng/ml) after incubation for another 3 h. **(B)** Effects of 3-h preincubation with degalactosylated whey protein (100 μg mg/ml) on mRNA expression of IL-1β induced by LPS (5 ng/ml) after incubation for another 3 h. **(C)** Effects of 3-h incubation with degalactosylated whey protein (100 μg mg/ml) on mRNA expression of TRL-4. **(D)** Effects of 3-h incubation with degalactosylated whey protein (100 μg mg/ml) on mRNA expression of MKP-1. Results are expressed as mean ± SE for 8–12. *p < 0.05, ^**^p < 0.01.

The increased mRNA expression of TNF-α and IL-1β in response to LPS (5 ng/ml) in RAW264.7 cells was significantly suppressed by the application of degalactosylated whey protein (100 μg/ml) to 75 and 79%, respectively, of the levels of cells treated with LPS alone, respectively, while degalactosylated whey protein itself did not change the mRNA expression of TNF-α and IL-1β in RAW264.7 cells (Figures 5A,B). In addition, the application of degalactosylated whey protein (100 μg/ml) to RAW264.7 cells induced a significant reduction in the mRNA expression of TLR4 to 80% of the control group (Figure 5C). Moreover, the application of degalactosylated whey protein (100 μg/ml) to RAW264.7 cells induced a significant increase in the mRNA expression of MKP-1 to 122% of the control group (Figure 5D).

## Discussion

The findings of the present study demonstrated that degalactosylated whey protein, but not intact whey protein, significantly suppressed the increase in plasma TNF-α and IL-1β levels following LPS administration in mice. In addition, following *in vitro* application of degalactosylated whey protein to mouse macrophage Raw264.7 cells, TLR4 mRNA was significantly decreased and MKP-1 mRNA was significantly increased. Moreover, degalactosylated whey protein significantly suppressed the increase in TNF-α and IL-1β mRNA induced by LPS application in Raw264.7 cells. These findings suggest that degalactosylated whey protein has anti-inflammatory activity.

Whey protein intake is beneficial for exercise performance and exercise enhancement. Whey protein also appears to be very promising as a functional food with several health benefits, such as antioxidant, antihypertensive, antitumor, antiviral, hypolipidemic, and antibacterial activity (2, 31). Further, whey protein is reported to exhibit several immunoregulatory actions (4–7). Animal studies demonstrated that whey protein reduces the production of proinflammatory cytokines like TNF-a, IL-6, and IL-1β, and increases the production of anti-inflammatory cytokines like IL-4 (8–11). Our findings of this study revealed that degalactosylated whey protein, but not intact whey protein, significantly reduced the increased plasma levels of TNF-α and IL-1β following LPS administration in mice. Protein glycosylation is a common post-translational modification of numerous important biological processes (12). Protein glycosylation plays a significant role in protein folding, targeted transport, cellular localization, and activity (32). On the other hand, deglycosylation converts biologically inactive proteins to biologically active proteins. For example, non-glycosylated V2 vasopressin receptors are fully active for ligand binding and signal transduction (21). Similarly, in the case of whey protein, the present study showed that galactosidase treatment converts inactive protein to active protein to suppress LPS responses. LPS has complex interactions with TLR4 on host immune cells including macrophages and microglia, where it induces the production of pro-inflammatory cytokines (33). These cytokines invoke a constellation of motivated behavioral adaptations, so-called sickness behavior (34). Sickness behaviors include loss of appetite and body weight, fatigue, withdrawal from normal social activities, altered cognition, hyperalgesia, and fever (35, 36). Sickness behaviors are well known to be due to the central actions of proinflammatory cytokines, such as TNF-α and IL-1β (37). In the present study, consistent with the previous reports (27, 34), LPS induced a marked decrease in body weight at 4 h after its administration in mice, which was significantly suppressed by the administration of degalactosylated whey protein, but not intact whey protein. Moreover, the present study showed that degalactosylated whey protein, but not intact whey protein, significantly decreased the enhancement of TNF-α and IL-1β production in response to LPS in *in vivo* and *in vitro* studies. Our findings suggest that degalactosylated whey protein attenuates sickness behavior.

We next tried to confirm the suppressive effects of degalactosylated whey protein on the mRNA expression of TNF-α and IL-1β using mouse macrophage Raw264 cells. Moreover, we examined the effects of degalactosylated whey protein on the expression of TLR4 and MKP-1 to elucidate the mechanisms underlying the anti-inflammatory activities of degalactosylated whey protein. Stimulation of TLR4 activates proinflammatory pathways and induces cytokine expression in a variety of cell types (38). Accordingly, suppression of TLR4 signaling could be therapeutically useful for suppressing an inappropriately overactive immune system (33). TLR4 antagonists decrease the production of pro-inflammatory cytokines in LPS-stimulated cultured human monocytes (33). The signaling transduction resulting from interactions between TLR4 and the mitogen-activated protein kinase (MAPK) family including the extracellular signal-regulated kinase 1/2 (ERK1/2), Jun N-terminal kinase (JNK), and p38 subfamilies, plays a pivotal role in the biosynthesis of proinflammatory cytokines in response to LPS in macrophages (38–42). MAPK phosphatase-1 (MKP-1) is an archetypal member of the dual specificity protein phosphatase family that dephosphorylates MAPK. MKP-1 is an anti-inflammatory phosphatase that negatively regulates MAPK activities (43, 44). Similarly, MKP-1-knockout mice showed the enhancement of plasma proinflammatory cytokine levels in response to LPS compared with wild-type mice, and, moreover, MKP-1 deficient macrophages exhibited enhanced production of proinflammatory cytokines compared with wild-type cells (45). MKP-1 upregulation in the central nervous system is also associated with reduced inflammation *via* endocannabinoid-mediated microglial expression (46). In the present study, in Raw264 cells, degalactosylated whey protein significantly suppressed TNF-α and IL-1β mRNA expression increased after LPS stimulation. Moreover, degalactosylated whey protein significantly decreased TLR4 expression and increased MKP-1 expression in Raw264 cells. Based on these findings, the anti-inflammatory actions of degalactosylated whey protein are attributed to deceased TLR4 expression and increased MKP-1 expression. These findings indicate that degalactosylated whey protein has potent immunoregulatory effects. However, the study on oral administration of whey proteins should be necessary to elucidate the role of whey protein supplementation in suppressing inappropriate overactivation of the immune response.

## Conclusion

The present findings revealed that degalactosylated whey protein has an anti-inflammatory effect in comparison to intact whey protein. The present data expand our knowledge about the role of degalactosylated whey protein in suppressing inappropriate overactivation of the immune response.

## Data Availability Statement

The original contributions presented in the study are included in the article/supplementary material, further inquiries can be directed to the corresponding author/s.

## Ethics Statement

The animal study was reviewed and approved by Akira Sano, President of Kagoshima University.

## Author Contributions

TI, NK, and GK performed the experiments, contributed to discussions, and wrote the manuscript. RN performed the experiments. AI contributed to discussions, reviewed, and edited the manuscript. NK was the guarantor of this work and, as such, had full access to all the data in this study and took responsibility for the integrity of the data and the accuracy of the data analysis. All authors contributed to the article and approved the submitted version.

## Conflict of Interest

The authors declare that the research was conducted in the absence of any commercial or financial relationships that could be construed as a potential conflict of interest.

## Publisher’s Note

All claims expressed in this article are solely those of the authors and do not necessarily represent those of their affiliated organizations, or those of the publisher, the editors and the reviewers. Any product that may be evaluated in this article, or claim that may be made by its manufacturer, is not guaranteed or endorsed by the publisher.
